# Global, regional, and national analyses of the burden of pancreatic cancer attributable to high fasting plasma glucose from 1990 to 2021: A longitudinal observational study

**DOI:** 10.1097/MD.0000000000048315

**Published:** 2026-04-17

**Authors:** Jiaqi Han, Qianping Liang, Yaqi Lv, Hongli Chen, Xiaoyu Lei, Chan Liao, Shaoqi Yang, Feifei Chu

**Affiliations:** a Department of Gastroenterology, Zhengzhou Central Hospital Affiliated to Zhengzhou University, Zhengzhou, China; b Henan Provincial Medicine Key Laboratory of Colorectal Cancer Diagnosis and Treatment, Zhengzhou, China; c Zhengzhou Key Laboratory of Colorectal Cancer Diagnosis, Treatment and Research, Zhengzhou, China.

**Keywords:** age-standardized mortality rate, disability-adjusted life years, GBD 2021, global burden of disease, high fasting plasma glucose, pancreatic cancer

## Abstract

High fasting plasma glucose (HFPG) is the second dominant metabolic risk factor contributing to the global burden of pancreatic cancer (PC). However, detailed investigations into the spatiotemporal patterns of PC burden attributable to HFPG remain limited. This study aims to assess global, regional, and national trends in PC mortality and disability-adjusted life years (DALYs) attributable to HFPG from 1990 to 2021. This longitudinal observational study was based on data from the global burden of disease 2021 study, covering data from 204 countries and territories. We extracted mortality, DALYs, age-standardized mortality rate (ASMR), and age-standardized DALY rate (ASDR) for PC attributable to HFPG. These metrics were stratified by sex, age group, country, and socio-demographic index (SDI). Temporal trends were evaluated using the estimated annual percentage change (EAPC) for ASMR and ASDR between 1990 and 2021. In 2021, an estimated 132,753 (95% uncertainty interval [UI]: 15,077–252,345) deaths and 2,751,644 (95% UI: 315,351–5,201,444) DALYs were attributable to HFPG, accounting for 40.9% and 39.3% of total PC-related deaths and DALYs, respectively. From 1990 to 2021, the number of HFPG-attributable PC deaths and DALYs increased by 234.1% and 209.7%, respectively. Substantial regional and national disparities were observed in the burden of PC attributable to HFPG. The highest ASMR and ASDR were recorded in high-SDI regions. Among global regions, East Asia reported the largest number of HFPG-attributable PC deaths and DALYs. The burden was also disproportionately higher among males and older adults. Notably, both ASMR and ASDR were significantly inversely correlated with EAPC. The global burden of PC attributable to HFPG has risen substantially over the past 3 decades, with marked regional and demographic disparities. These findings underscore the urgent need for glycemic control strategies and nutrition-based public health interventions to reduce HFPG-related cancer burden, particularly among high-risk populations.

## 1. Introduction

Pancreatic cancer (PC) is the 12th most common type of cancer. It is characterized by high malignancy, rapid progression, and poor prognosis compared to other major cancers. Early symptoms are often subtle, making diagnosis challenging, and as the disease advances, it spreads to nearby organs, leading to severe complications and poor survival outcomes. PC is the seventh leading cause of cancer-related deaths worldwide, underscoring its global public health burden.

High fasting plasma glucose (HFPG) is the top accelerating factor for global cancer mortality^[1]^ and the second leading metabolic risk factor for PC mortality after high BMI.^[2]^ The pathophysiological mechanisms underlying the association between PC and hyperglycemia remain incompletely elucidated. However, it is widely recognized that chronic inflammation, insulin resistance, hyperinsulinemia, elevated glucose levels, and insulin-like growth factor (IGF) signaling axis abnormalities all play a part in the onset and advancement of PC when hyperglycemia is present.^[3]^ Notably, PC and diabetes exhibit a bidirectional relationship: insulin resistance associated with diabetes leads to compensatory hyperinsulinemia and persistent hyperglycemia, while concomitant inflammation and obesity further amplify the oncogenic risk of PC.^[4]^ In addition, abnormal expression of tumor suppressors and oncogenes has been shown in experiments to upregulate glucose transporters and glycolytic enzymes, facilitating their translocation to the plasma membrane and enhancing glucose uptake by tumor cells.^[5]^ Despite diabetes increasing PC risk,^[6]^ only 2.7% of PC prevention studies focus on metabolic interventions versus 61% on genetics. While Type 2 Diabetes Mellitus dietary prevention reduces PC risk,^[7]^ spatiotemporal analyses of HFPG-attributable PC mortality remain limited.^[8–10]^

Recent studies have used Global Burden of Disease (GBD) data to assess the global, regional, and national burden of PC and its risk factors, identifying smoking, HFPG, high BMI, alcohol use, and dietary risks as the main contributors to PC-related disability-adjusted life years (DALYs).^[11]^ One prior study^[12]^ analyzed the impact of elevated fasting glucose on PC in China from 1990 to 2021, but was limited to a single country. To date, no global analysis has comprehensively evaluated the burden and trends of PC attributable to HFPG. Accordingly, the present study was to assess the disease burden of PC related to HFPG at global, regional, and national levels using the most up-to-date estimates from the GBD 2021 study. In addition, the estimated annual percentage change (EAPC) was calculated to assess temporal trends in age-standardized rate (ASR) over the study period. The study’s results offer important insights into HFPG’s epidemiological role and can guide efforts to lower PC incidence and influence.

## 2. Materials and techniques

### 2.1. Source of data

All data were sourced from the GBD 2021 study, which applies a Bayesian hierarchical model (DisMod-MR 2.1) to integrate multi-source data and estimate values for missing regions.^[13]^ In low-socio-demographic index (SDI) areas with limited data, model covariates (e.g., healthcare access, cause-of-death coding quality) may introduce bias; this is addressed by reporting 95% UI. Using the Global Health Data Exchange (GHDx) platform (http://ghdx.healthdata.org/gbd-results-tool), we extracted estimates of HFPG-attributable PC deaths, DALYs, age-standardized mortality rate (ASMR), and age-standardized DALY rate (ASDR) for 204 countries and territories from 1990 to 2021. Data were stratified by sex, age, GBD region, and SDI level. Search terms included “pancreatic cancer,” “high fasting plasma glucose,” “death,” and “DALYs,” with time defined as “1990 to 2021” and metrics as “number,” “percent,” and “rate.”

### 2.2. Definitions

The GBD 2021 study defines fasting blood glucose ≥ 5.5 mmol/L as the risk exposure threshold.^[14]^ DALYs measure total healthy years lost from illness onset to death. ASR, adjusted to a standard population structure, enable comparisons of mortality and DALYs across regions and time by removing age distribution effects. The study also examined the link between disease burden and the SDI, a composite of income, fertility, and education. The SDI is a composite indicator used in GBD studies to approximate a location’s level of socioeconomic development. It is based on 3 covariates: lag-distributed income per capita, average educational attainment in the population aged ≥ 15 years, and total fertility rate under age 25 years. These covariates were selected because they capture complementary dimensions of development – economic productivity, human capital, and demographic transition – and together explain much of the variation in health outcomes across locations. Data on income and fertility were primarily obtained from the World Bank, the United Nations Population Division, and national statistical offices, while education data were derived from the Institute for Health Metrics and Evaluation (IHME) and international census/household survey databases. For locations or years with incomplete data, GBD applied spatiotemporal Gaussian process regression to generate estimates, ensuring comparability across all 204 countries and territories from 1990 to 2021. Based on SDI, the 204 countries and territories were grouped into 5 levels: low (<0.45), low-middle (≥0.45–<0.61), middle (≥0.61–<0.69), high-middle (≥0.69–<0.80), and high (≥0.80).

### 2.3. Analysis of statistics

The number of fatalities, DALYs, ASMR, and ASDR were used to calculate the burden of PC due to HFPG by sex, age group, GBD area, country, and SDI quintile. The ASR was calculated using the formula below:


$$\begin{array}{l} ASR=\left(\frac{\Sigma_{i}a_{i}w_{i}}{\Sigma_{i}w_{i}}\right)\times 100,000 \end{array}$$

where A denotes the total number of age groupings, *w*_*i*_ is the number of individuals (or the allocated weight) in that age group based on the selected standard population, and *a*_*i*_ is the age-specific rate in the *i*-th age group. To account for variations in population age distribution, all rates were expressed per 100,000 people. The weighting was derived from the global age-standard population defined in the GBD 2021 study, rather than from the 2021 world population distribution.

To evaluate temporal trends in ASR, the EAPC was calculated using linear regression, with the natural logarithm of ASR (ASMR or ASDR) regressed against year (*y* = α+β*x*+ε), where *x* represents year, *y* is the ln-transformed ASR, β indicates the annual change, and ε is the error term. The EAPC was determined to be:

EAPC = 100×(exp(β)−1)

The 95% confidence interval (CI) for the EAPC was obtained from the regression model. An ASR was considered increasing if both the EAPC and its 95% CI were >0, decreasing if both were <0, and stable if the CI included 0.

Hierarchical cluster analysis was employed to categorize the 204 countries and territories into 4 groups based on the direction and magnitude of EAPC values: stable, minor increase, significant increase, and decrease. To explore factors influencing EAPC, Pearson correlation was used to assess its association with baseline ASRs in 1990 and 2021, given its suitability for linear relationships between continuous variables. All analyses were performed using R version 4.4.3, with *P* < .05 considered statistically significant.

In low-resource settings, incomplete case ascertainment and limited diagnostic capacity may result in underreporting of pancreatic cancer outcomes. To address such gaps, the GBD framework applies multiple statistical approaches, including spatiotemporal Gaussian process regression and Bayesian meta-regression (DisMod-MR), which borrow strength across time, geography, and covariates to generate consistent estimates even when primary data are sparse. For SDI covariates, data on income and fertility were obtained from the World Bank, the UN Population Division, and national statistical offices, while education data were derived from IHME and international census or household survey databases. Where information was missing, the same standardized modeling approaches were applied to ensure comparability and full coverage across all 204 countries and territories.

## 3. Results

### 3.1. PC trends worldwide linked to HFPG

**Figure 1.**
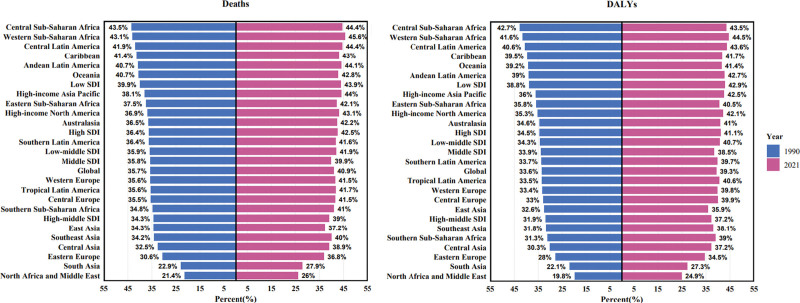
Proportion of high fasting plasma glucose cancer deaths and DALYs attributable to high fasting plasma glucose globally and in 27 GBD regions in 1990 and 2021. DALYs = disability adjusted life-years, GBD = Global Burden of Disease Study.

**Figure 2.**
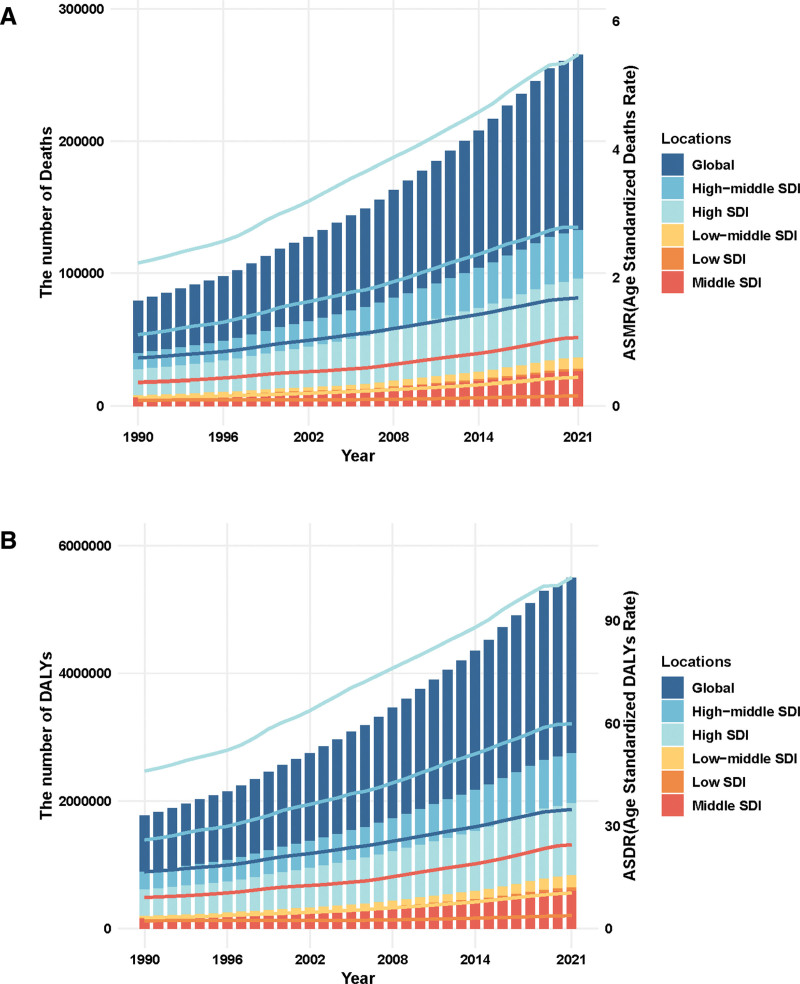
Number and rate of pancreatic cancer deaths (A) and DALYS (B) attributable to high fasting plasma glucose from 1990 to 2021 by SDI level. The bars represent the number of pancreatic cancer deaths (A) and DALYS (B) attributable to high fasting plasma glucose from 1990 to 2021 colored by SDI level. The line represents the mean ASMR (A) and ASDR (B) (per 100,000) attributable to high fasting plasma glucose at the global level. The shaded area represents the 95% UI for the mean rate. ASMR = age-standardized mortality rate, ASDR = age-standardized DALY rate, DALYs = disability-adjusted life-years, SDI = socio-demographic index, UI = uncertainty interval.

Figure 1 shows that in 2021, the global number of PC-related deaths and DALYs attributable to HFPG was estimated at 132,753 (95% UI: 15,077–252,345) and 2,751,644 (95% UI: 315,351–5201,444), respectively, accounting for 40.9% of all PC-related deaths and 39.3% of total DALYs. Between 1990 and 2021, the number of deaths due to PC associated with HFPG increased by 234.1%, rising from 39,731 (95% UI: 4532–77,922) to 132,753 (95% UI: 150.77–252,345). Similarly, the DALYs count rose by 209.7%, from 888,340 (95% UI: 101,475–1748,791) to 2,751,644 (95% UI: 315,351–5,201,444). In 2021, the ASDR and ASMR were 31.7 (95% UI: 3.63–59.9) and 1.57 (95% UI: 0.18–2.98) per 100,000 population, respectively. Both global ASMR and ASDR for PC attributable to HFPG demonstrated a clear upward trend over the study period, with EAPC of 1.34% (95% CI: 1.27–1.41) and 1.22% (95% CI: 1.16–1.28), respectively (Table 1; Fig. 2).

**Table 1:** Global burden of pancreatic cancer in 1990 and 2021 for both sexes and all locations, with EAPC.

	1990				2021				1990–2021		
	Death cases No. ×10^2^ (95% UI)	ASMR per 100,000 No. (95% UI)	DALY cases No. ×10^2^ (95%UI)	ASDR per 100,000 No. (95% UI)	Death cases No. ×10^2^ (95% UI)	ASMR per 100,000 No. (95% UI)	DALY cases No. ×10^2^ (95% UI)	ASDR per 100,000 No. (95% UI)	EAPC of ASMR No. (95% CI)	EAPC of ASDR No. (95% CI)	
Global	397.31 [45.32–779.22]	1.08 [0.12–2.11]	8883.4 [1014.75–17,487.91]	22.64 [2.58–44.6]	1327.53 [150.77–2523.45]	1.57 [0.18–2.98]	27,516.44 [3153.51–52,014.44]	31.7 [3.63–59.9]	1.34 [1.27–1.41]		1.22 [1.16–1.28]
Sex											
Female	188.49 [21.36–365.6]	0.92 [0.1–1.79]	3871.23 [441.76–7602.23]	18.31 [2.09–35.89]	612.25 [69.21–1177.41]	1.31 [0.15–2.52]	11,785.74 [1349.34–22,436.69]	25.35 [2.9–48.26]	1.23 [1.16–1.29]		1.12 [1.07–1.17]
Male	208.82 [23.95–409.75]	1.27 [0.15–2.49]	27.47 [3.15–53.86]	15,730.7 [1804.17–29,846.56]	715.28 [81.55–1349.45]	1.86 [0.21–3.52]	15,730.7 [1804.17–29,846.56]	38.69 [4.43–73.29]	1.42 [1.34–1.51]		1.29 [1.21–1.36]
SDI region											
High SDI	195.93 [22.59–373.89]	1.73 [0.2–3.3]	4053.92 [468.84–7790.29]	36.45 [4.21–70.16]	599.66 [69.63–1119.88]	2.66 [0.31–4.96]	11,230.97 [1320.09–20,812.18]	53.8 [6.34–99.48]	1.57 [1.47–1.66]		1.42 [1.32–1.52]
High-middle SDI	118 [13.3–234.07]	1.23 [0.14–2.45]	2761.26 [310.48–5480.71]	27.4 [3.09–54.29]	363.3 [40.93–693.94]	1.81 [0.2–3.47]	7809.81 [887.74–14,957.62]	38.86 [4.42–74.42]	1.42 [1.33–1.52]		1.28 [1.2–1.36]
Middle SDI	63.36 [7.15–127.89]	0.69 [0.08–1.38]	1574.44 [178.76–3178.6]	15.17 [1.71–30.57]	260.35 [28.73–507.25]	1 [0.11–1.96]	6009.69 [671.82–11,688.44]	21.88 [2.44–42.52]	1.3 [1.24–1.35]		1.25 [1.2–1.3]
Low-middle SDI	14.74 [1.58–29.07]	0.27 [0.03–0.53]	363.56 [38.99–721.8]	6.01 [0.65–11.89]	84.87 [9.37–169.29]	0.63 [0.07–1.25]	2006.48 [223–4032.52]	13.91 [1.54–27.88]	2.88 [2.84–2.93]		2.84 [2.81–2.88]
Low SDI	4.65 [0.48–9.28]	0.23 [0.02–0.47]	116.32 [12.11–232.16]	5.19 [0.54–10.36]	17.72 [1.85–34.75]	0.4 [0.04–0.79]	425.77 [45.19–836.53]	8.6 [0.9–16.87]	1.75 [1.58–1.91]		1.6 [1.44–1.77]
GBD regions											
Tropical Latin America	9.36 [0.96–18.85]	1.13 [0.12–2.26]	217.5 [22.57–436.69]	24.14 [2.5–48.62]	42.37 [4.83–80.31]	1.67 [0.19–3.17]	942.94 [108.87–1779.57]	36.35 [4.19–68.74]	1.51 [1.43–1.59]		1.53 [1.46–1.6]
Eastern Sub-Saharan Africa	1.37 [0.13–2.93]	0.23 [0.02–0.49]	32.15 [3.12–68.76]	4.59 [0.44–9.78]	5.44 [0.52–11.93]	0.39 [0.04–0.83]	127.21 [12.44–279.1]	8.01 [0.77–17.58]	1.7 [1.59–1.82]		1.71 [1.6–1.83]
Southern Latin America	8.01 [0.88–15.57]	1.75 [0.19–3.41]	171.93 [19.3–333.09]	36.73 [4.11–71.08]	22.68 [2.63–43.35]	2.53 [0.29–4.82]	460.82 [53.87–877.73]	52.55 [6.15–100.13]	1.39 [1.24–1.53]		1.32 [1.17–1.47]
Andean Latin America	1.36 [0.13–2.92]	0.73 [0.07–1.58]	29.79 [2.87–62.49]	15.1 [1.46–31.83]	8.03 [0.84–16.72]	1.41 [0.15–2.94]	165.35 [17.86–342.53]	28.39 [3.05–58.76]	2.28 [2.12–2.45]		2.13 [1.98–2.28]
Australasia	3.13 [0.38–5.71]	1.31 [0.16–2.39]	65.31 [7.92–118.91]	27.49 [3.33–50.13]	10.67 [1.34–18.7]	1.88 [0.24–3.29]	203.43 [25.71–356.21]	38.31 [4.82–67.46]	1.37 [1.28–1.46]		1.3 [1.21–1.39]
Caribbean	3.28 [0.36–6.46]	1.31 [0.14–2.57]	67.2 [7.42–132.79]	26.07 [2.88–51.47]	8.62 [1–16.93]	1.59 [0.19–3.13]	184.28 [21.52–363.37]	34.11 [3.99–67.27]	1.1 [0.93–1.26]		1.29 [1.14–1.44]
Central Asia	1.88 [0.18–3.88]	0.41 [0.04–0.86]	47.41 [4.68–96.31]	9.9 [0.98–20.14]	8.22 [0.85–16.7]	1.08 [0.11–2.21]	201.87 [21.2–405.91]	24.18 [2.52–48.91]	3.57 [3.29–3.85]		3.25 [2.99–3.51]
Central Europe	25.81 [2.97–49.85]	1.74 [0.2–3.36]	587.14 [67.22–1138.37]	38.42 [4.39–74.52]	66.2 [7.97–123.71]	2.84 [0.34–5.3]	1365.87 [166.21–2547.04]	61.33 [7.48–114.32]	1.65 [1.54–1.76]		1.59 [1.46–1.72]
Central Latin America	10.14 [1.18–19.17]	1.34 [0.16–2.53]	234.12 [27.64–442.1]	28.65 [3.37–54.13]	37 [4.49–71]	1.52 [0.18–2.91]	836.42 [103.71–1596.44]	33.19 [4.1–63.37]	0.13 [0.02–0.24]		0.19 [0.08–0.31]
Central Sub-Saharan Africa	0.94 [0.11–1.82]	0.49 [0.06–0.95]	24.79 [2.85–48.28]	10.98 [1.25–21.13]	2.98 [0.35–6.06]	0.63 [0.07–1.29]	78.41 [9.31–159.63]	14.21 [1.68–28.99]	0.77 [0.51–1.03]		0.76 [0.5–1.02]
East Asia	71.99 [8.1–143.77]	0.9 [0.1–1.79]	1865.6 [207.8–3740.76]	20.79 [2.33–41.67]	273.46 [30.57–520.09]	1.26 [0.14–2.4]	6292.47 [715.84–11,974.19]	28 [3.17–53.31]	1.27 [1.09–1.45]		1.16 [1.02–1.31]
Eastern Europe	25.42 [2.67–52.77]	0.9 [0.09–1.87]	625.13 [65.87–1293.71]	21.78 [2.3–45.02]	59.48 [6.3–118.28]	1.64 [0.17–3.27]	1330.11 [141.46–2636.03]	37.44 [3.98–74.41]	2.02 [1.81–2.23]		1.73 [1.54–1.92]
High-income Asia Pacific	40.17 [4.97–73.09]	2.06 [0.25–3.73]	851.31 [104.94–1565.94]	41.94 [5.17–76.94]	133.35 [16.34–242.22]	2.46 [0.3–4.47]	2194.17 [270.04–3972.22]	46.79 [5.76–84.22]	0.59 [0.55–0.62]		0.34 [0.29–0.39]
High-income North America	68.1 [8.19–127.64]	1.88 [0.23–3.52]	1423.14 [171.95–2659.72]	40.84 [4.93–76.16]	220.15 [27.01–401.49]	3.19 [0.39–5.81]	4376.31 [543.47–7888.73]	66.17 [8.22–119.46]	1.96 [1.77–2.15]		1.77 [1.6–1.94]
North Africa and Middle East	8.85 [0.94–17.8]	0.6 [0.06–1.19]	214.48 [22.59–430.21]	12.92 [1.37–25.95]	61 [6.96–115.38]	1.5 [0.17–2.85]	1433.51 [164.97–2713.38]	31.87 [3.65–60.17]	3.45 [3.23–3.68]		3.33 [3.12–3.54]
Oceania	0.14 [0.02–0.28]	0.58 [0.06–1.14]	3.63 [0.42–7.22]	12.61 [1.42–24.85]	0.52 [0.06–1.02]	0.82 [0.1–1.58]	13.5 [1.59–26.72]	18.08 [2.13–35.46]	1.23 [1.16–1.3]		1.26 [1.18–1.34]
South Asia	11.34 [1.26–22.07]	0.22 [0.02–0.43]	291.5 [32.41–565.41]	5.01 [0.56–9.74]	60.04 [6.59–113.98]	0.43 [0.05–0.82]	1426.74 [158.22–2719.51]	9.56 [1.06–18.22]	2.04 [1.87–2.21]		1.91 [1.73–2.08]
Southeast Asia	9.2 [0.92–19.49]	0.41 [0.04–0.88]	220.18 [22.11–465.36]	8.86 [0.89–18.69]	52.89 [5.33–109.04]	0.88 [0.09–1.82]	1223.92 [124.31–2542]	18.66 [1.89–38.58]	2.42 [2.34–2.49]		2.34 [2.27–2.4]
Southern Sub-Saharan Africa	1.85 [0.17–3.92]	0.79 [0.07–1.68]	40.35 [3.91–85.58]	15.63 [1.5–33.07]	8.45 [0.78–17.24]	1.64 [0.15–3.34]	194.92 [18.31–399.85]	34.31 [3.19–70.19]	2.64 [2.29–2.99]		2.86 [2.49–3.22]
Western Europe	93.71 [10.04–186.83]	1.54 [0.17–3.07]	1841.01 [197.39–3660.91]	31.5 [3.38–62.55]	237.75 [24.71–473.68]	2.33 [0.24–4.63]	4271.64 [447.47–8455.71]	46.37 [4.88–91.67]	1.53 [1.47–1.6]		1.45 [1.37–1.53]
Western Sub-Saharan Africa	1.29 [0.13–2.6]	0.17 [0.02–0.34]	29.73 [3.11–59.74]	3.49 [0.36–7.05]	8.23 [0.85–17.07]	0.5 [0.05–1.02]	192.57 [20–396.94]	10.31 [1.06–21.36]	3.92 [3.79–4.05]		3.88 [3.76–4.01]

ASDR = age-standardized DALY rate, ASMR = age-standardized mortality rate, DALYs = disability-adjusted life-years, EAPC = estimated annual percentage change, SDI = socio-demographic index, UI = uncertainty interval.

### 3.2. Regional differences in PC caused by HFPG

Table 1 shows that in 2021, the high SDI regions had the highest number of PC-related deaths [59,966 (95% UI: 6,963–111,988)] and DALYs [1,123,097 (95% UI: 132,009–2,081,218)] due to HFPG. In contrast, regions with the lowest SDI recorded the fewest PC-related deaths and DALYs, estimated at 1772 (95% UI: 185–3475) and 94,294 (95% UI: 10,887–177,957), respectively. By comparison, high-SDI regions exhibited the highest ASMR and ASDR, with values of 2.66 (95% CI: 0.31–4.96) and 53.8 (95% CI: 6.34–99.48) per 100,000 population, respectively. From 1990 to 2021, the ASMR and ASDR of the low, low-middle, middle, high middle and high SDI regions had gradually increased.

Geographically, East Asia had the highest number of PC-related deaths [27,346 (95% UI: 3057–52,009)] and DALYs [133,011 (95% UI: 14,146–263,603)] in 2021 associated with HFPG. In 2021, Eastern Sub-Saharan Africa reported the lowest ASMR and ASDR, at 0.39 and 8.01 per 100,000 population, respectively, whereas the highest values were observed in High-income North America, with an ASMR of 3.19 and an ASDR of 66.67 per 100,000. Over the period from 1990 to 2021, Western Sub-Saharan Africa experienced the most pronounced increases in ASMR and ASDR, with estimated annual percentage changes of 3.92 (95% CI: 3.79–4.05) and 3.88 (95% CI: 3.76–4.01), respectively. However, Central Latin America had the smallest increases in ASMR and ASDR [0.13 (95% CI: 0.02–0.24)] and [0.19 (95% CI: 0.08–0.31)] (Table 1). In 1990, the highest proportions of PC-related deaths and DALYs attributable to HFPG were observed in Central Sub-Saharan Africa, Western Sub-Saharan Africa, and Central Latin America. In contrast, North Africa, the Middle East, South Asia, and Eastern Europe exhibited the lowest attributable proportions. The disparity in HFPG-related PC burden across these regions in 1990 was approximately two-fold. Similar regional patterns were observed in 2021, indicating persistent inequalities in the proportion of PC burden attributable to HFPG (Fig. 1).

### 3.3. PC burden at the national level due to HFPG

**Figure 3.**
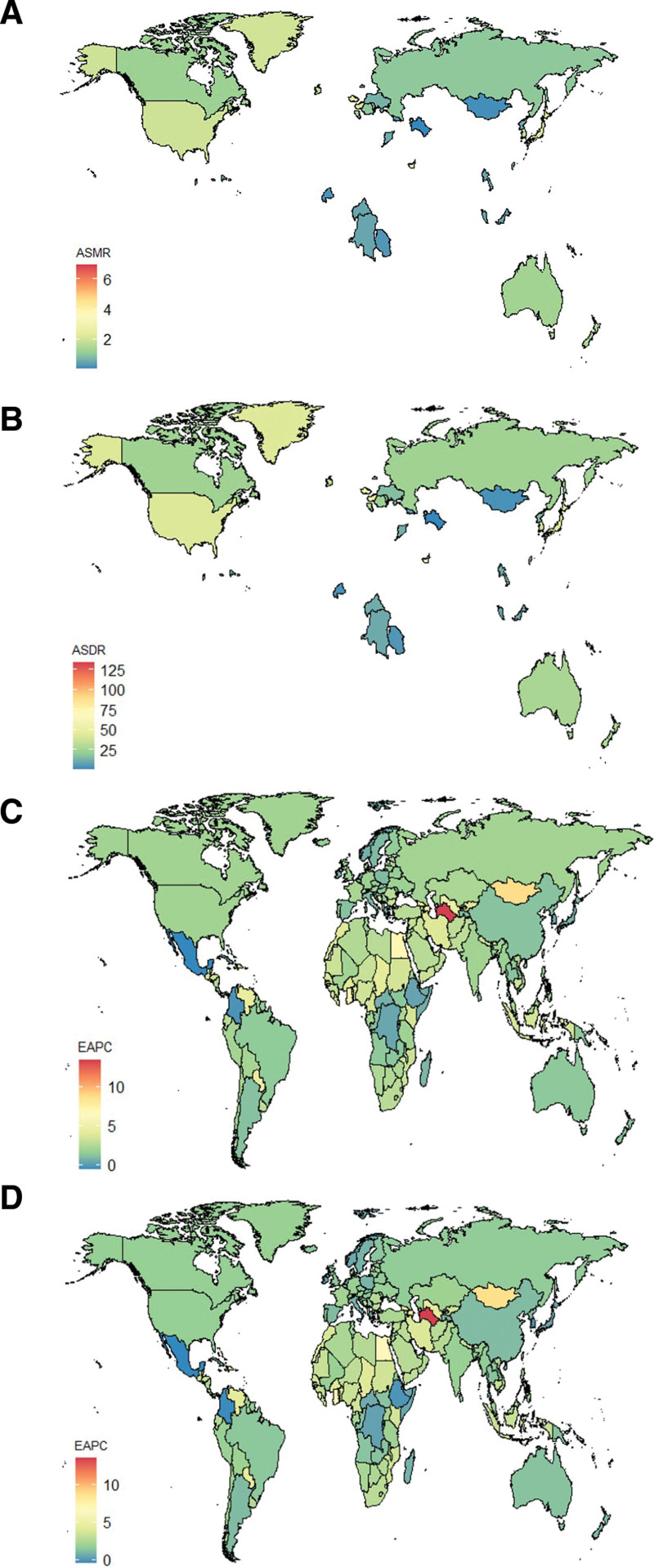
The spatial distribution of the pancreatic cancer ASMR (A) and ASDR (B) attributable to high fasting plasma glucose in 2021, and the EAPC in pancreatic cancer ASMR (C) and ASDR (D) attributable to high fasting plasma glucose. ASMR = age-standardized mortality rate, ASDR = age-standardized DALY rate, EAPC = estimated annual percentage change.

China had the greatest number of PC fatalities and DALYs linked to HFPG at the national level in 2021 [26,256 (95% UI: 2944–50,078) and 605,286 (95% UI: 69,071–1,158,558, respectively], followed by United States of America [20,424 (95% UI: 2523–36,719) and 408,621 (95% UI: 51,103–731,239), respectively], and Japan [11,350 (95% UI: 1384–20,309) and 180,382 (95% UI: 22,117–323,472), respectively]. Also in 2021, In the United Arab Emirates, the Czech Republic and Palau had the highest ASMR and ASDR due to HFPG (Fig. 3(A, B)). The greatest increases in ASMR were observed in Turkmenistan, Cabo Verde, and Mongolia, with EAPC of 13.46 (95% CI: 11.79–15.15), 9.05 (95% CI: 7.31–10.82), and 8.84 (95% CI: 8.12–9.57), respectively. A similar pattern was found for ASDR, where Turkmenistan, Cabo Verde, and Mongolia again showed the most substantial increases, with EAPCs of 13.53 (95% CI: 11.82–15.26), 9.02 (95% CI: 7.26–10.80), and 8.79 (95% CI: 8.06–9.52), respectively. With EAPCs of −0.58 (95% CI: −0.68–−0.48) and −0.44 (95% CI: −0.54–−0.34), respectively, Mexico, on the other hand, showed the most noticeable drops in both ASMR and ASDR (Fig. 3(C, D); Tables S1–S6, Supplemental Digital Content, https://links.lww.com/MD/R672).

### 3.4. Global PC load by sex and age related to HFPG

Globally, the burden of PC due to HFPG is still influenced by sex differences, with males bearing a greater disease burden, particularly in older age groups. In 2021, the number of PC-related deaths and DALYs attributable to HFPG was higher in males than in females, with 71,528 (95% UI: 8155–134,945) deaths and 1,573,070 DALYs (95% UI: 180,417–2,984,656) among males, compared to 61,225 (95% UI: 6921–117,741) deaths and 1,178,574 DALYs (95% UI: 134,934–2,243,669) among females (Table 1). Correspondingly, in 2021, the ASMR per 100,000 population was higher in males [2.56; 95% UI: 1.60–3.51] than in females [1.70; 95% UI: 1.07–2.28], and the ASDR was similarly elevated in males [1.86; 95% UI: 0.21–3.52] compared to females [1.31; 95% UI: 0.15–2.52].

**Figure 4.**
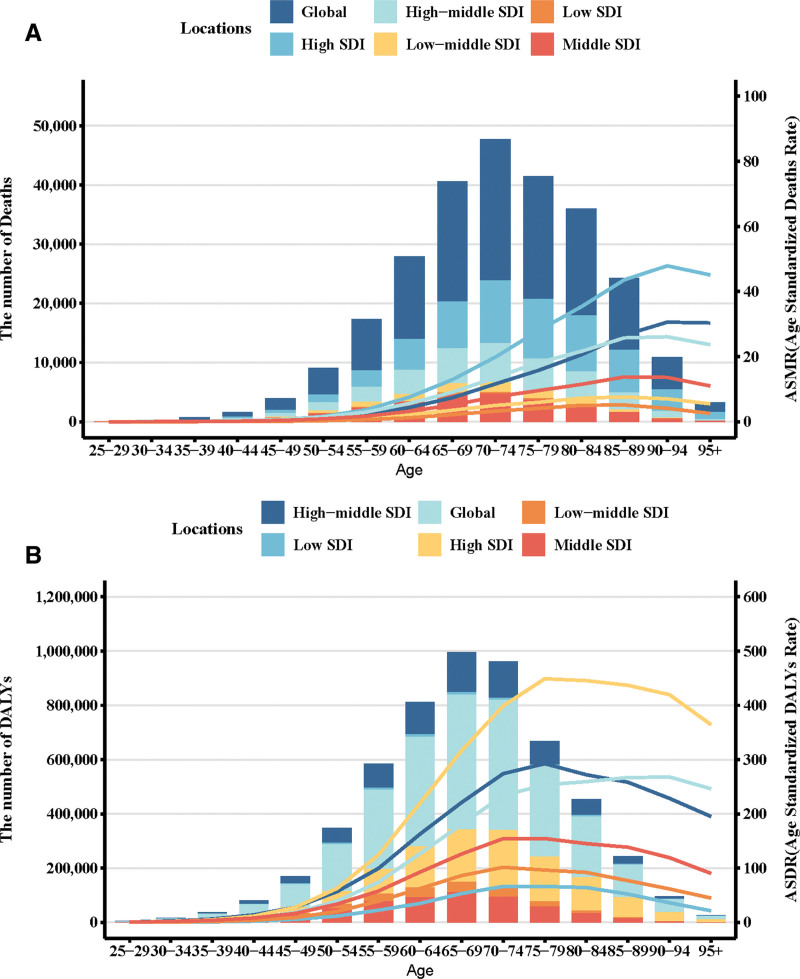
Number and rate of pancreatic cancer deaths (A) and DALYs (B) attributable to high fasting plasma glucose by age group and SDI level in 2021. The bars represent the number of pancreatic cancer deaths (A) and DALYs (B) attributable to high fasting plasma glucose colored by SDI level. The line represents the mean ASMR (A) and ASDR (B) (per 100,000) attributable to high fasting plasma glucose at the global level. The shaded area represents the 95% UI for the mean rate. DALYs = disability-adjusted life-years, ASMR = age-standardized mortality rate, ASDR = age-standardized DALY rate, SDI = sociodemographic index, UI = uncertainty interval.

**Figure 5.**
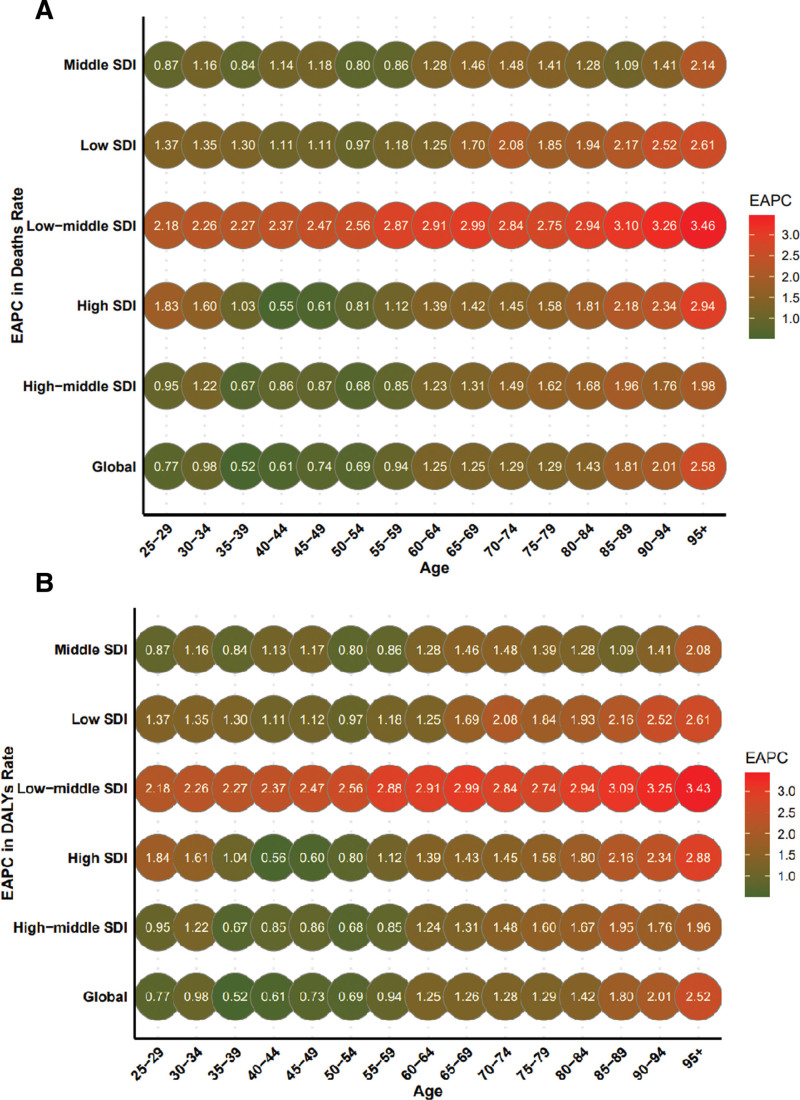
Annual percentage change in mortality (A) and DALYs (B) between 1990 and 2021 by age group and region. EAPC = estimated annual percentage change, DALYs = disability-adjusted life-years, SDI = socio-demographic index.

Between 1990 and 2021, the average annual increase in ASMR and ASDR attributable to HFPG was 1.42% (95% CI: 1.34–1.51) and 1.29% (95% CI: 1.21–1.36), respectively, for males, and 1.23% (95% CI: 1.16–1.29) and 1.12% (95% CI: 1.07–1.17), respectively, for females. In 2021, the ASMR increased with age, peaking in individuals aged 70 to 74 years (Fig. 4A). In contrast, the ASDR reached its highest level in the 90 to 94 age group and then declined with further age, while the highest number of DALYs occurred in the 65 to 69 age group (Fig. 4B). From 1990 to 2021, global ASMR increased across all age groups. The most rapid increase was observed among individuals aged 95 years and older, whereas the 25 to 29 age group exhibited the slowest growth. Over the same period, ASMR increased across all SDI quintiles – including low, low-middle, middle, high-middle, and high SDI regions – with the highest EAPC observed in low-middle SDI settings (Fig. 5A). Trends in ASDR mirrored those of ASMR, with comparable EAPCs across regions (Fig. 5B).

Hierarchical cluster analysis identified 123 countries or territories – most notably New Zealand, China, and Tonga – as exhibiting stable ASMR trends (“remained stable” cluster). In contrast, 74 countries or territories, including Monaco, Portugal, and Kuwait, were classified into the “increasing” trend group. Notably, Turkmenistan was the only country categorized as showing a “significant decrease” in ASMR attributable to HFPG. The remaining 6 nations or territories, including Ghana, Lesotho, Egypt, were classified as “decrease” (Fig. S1, Supplemental Digital Content, https://links.lww.com/MD/R672).

### 3.5. Factors associated with the burden of PC burden attributable to HFPG

**Figure 6.**
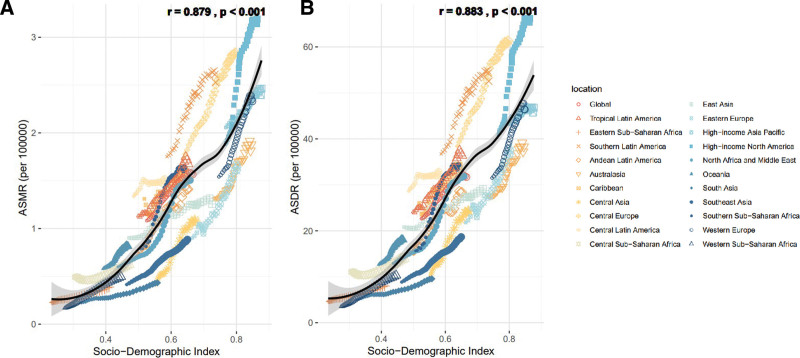
Correlation between high fasting plasma glucose-attributable high fasting plasma glucose cancer in ASMR (A) or ASDR (B) and SDI globally in 21 GBD regions between 1990 and 2021. ASMR = age-standardized mortality rate, ASDR = age-standardized DALY rate, GBD = global burden of disease study.

Overall, from 1990 to 2021, ASMR and ASDR of PC caused by HFPG were significantly positively correlated with SDI (*R* = 0.879 and 0.883, *P* < .001). With the increase of SDI, the mortality rate and burden of PC accelerated, especially after the SDI > 0.6, and the ASMR and ASDR were at the highest level in the world in high-income North America, East Asia, and high-income Asia-Pacific (Fig. 6(A,B)).

**Figure 7.**
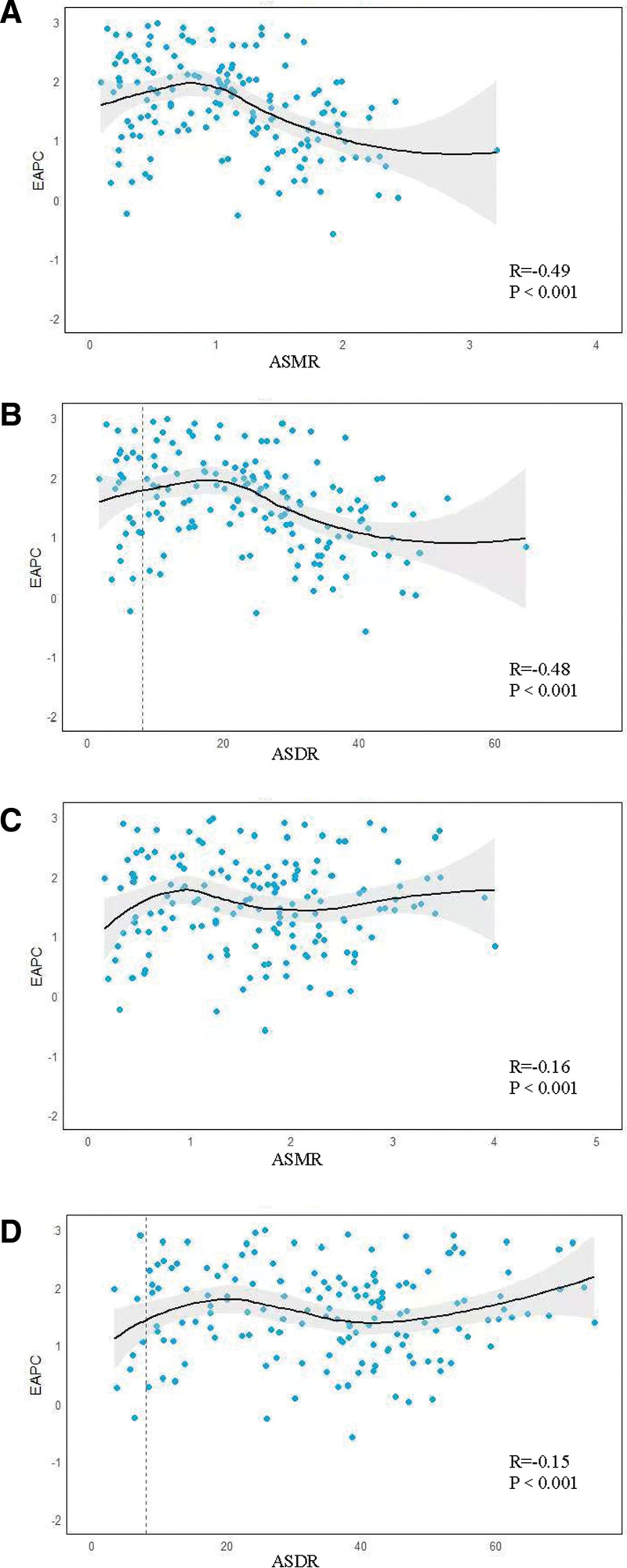
Correlation between EAPC and ASMR (A), ASDR (B) in 1990. Correlation between EAPC and ASMR (C), ASDR (D) in 2021. EAPC = estimated annual percentage change, ASMR = age-standardized mortality rate, ASDR = age-standardized DALY rate.

From 1990 to 2021, ASMR in PC caused by HFPG showed a significant negative association with ASDR and EAPC worldwide, indicating that the downward trend of EAPC was more obvious in regions with higher ASMR or ASDR. The correlation between ASMR and ASDR and EAPC in 1990 was strong (*R* = −0.49 and −0.48, *P* < .001) (Fig. 7(A, B)), suggesting a more significant association between early disease burden and rate of improvement. The weakened correlation between ASMR and ASDR and EAPC in 2021 (*R* = −0.16 and −0.15, *P* < .001) may suggest a narrowing of differences in the rate of improvement between regions with the spread of health interventions globally (Fig. 7(C, D)).

## 4. Discussion

This analysis provides a comprehensive overview of global, regional, and national epidemiological trends in PC due to HFPG. These findings fill a key knowledge gap on HFPG-attributable PC burden, emphasizing the need for stronger glycemic control and guiding metabolic and dietary interventions to curb PC-related mortality and disability worldwide.

At a global level, in 2021, HFPG was responsible for 40.9% of PC deaths and 39.3% of DALYs, with a significant increase in both HFPG-linked PC deaths and DALYs from 1990 to 2021, rising by 234.1% and 209.7%, respectively. This proportion may be explained by the parallel global increase in diabetes prevalence and population aging,^[15]^ which expand the pool of individuals at risk for HFPG-related pancreatic cancer. Lifestyle transitions characterized by sedentary behavior, rising obesity rates, and greater consumption of calorie-dense, high-fat, and high-sugar diets have further aggravated hyperglycemia and insulin resistance worldwide.^[16,17]^ From a biological perspective, chronic hyperglycemia promotes pancreatic carcinogenesis through sustained hyperinsulinemia, activation of the IGF-1 pathway, and induction of systemic inflammation and oxidative stress.^[18,19]^ In high-SDI regions, advances in diagnostic capacity and reporting systems may also contribute to higher observed mortality, whereas in low-SDI settings, under-detection may mask the true magnitude of the burden.^[20]^ Together, these demographic, behavioral, biological, and health system factors contextualize why HFPG accounts for such a large and growing share of the global pancreatic cancer burden.

While global estimates underscore the growing burden of pancreatic cancer attributable to HFPG, these overall patterns mask pronounced regional and socioeconomic disparities, highlighting the importance of examining trends across different SDI strata. This study examined disparities in the burden of PC attributable to HFPG across countries with different levels of SDI. From 1990 to 2021, high-SDI regions consistently exhibited higher ASMR and ASDR for PC associated with HFPG. These elevated rates may be partly explained by demographic and epidemiological factors prevalent in high-SDI settings, including aging populations, hereditary susceptibility, and long-term exposure to modifiable risk factors such as obesity, alcohol consumption, high-calorie diets, and malnutrition. Particularly, it has been suggested that the Western dietary pattern, which is marked by a high consumption of red and processed meats, refined cereals, and beverages with added sugar, promotes systemic inflammation, which may be a factor in the development of pancreatic cancer.^[21]^ Advances in screening and diagnostic technologies, together with improved healthcare access and reporting systems, have likely increased case detection in high-SDI regions. While these developments enhance surveillance, they may also contribute to inflated ASMR estimates, potentially overestimating the true mortality burden attributable to HFPG.

Interestingly, our analysis revealed more pronounced upward trends in ASDRs in low-SDI, low-middle-SDI, and middle-SDI regions over the same period. Notably, in the Asia-Pacific region, especially in countries undergoing rapid socioeconomic transitions, the burden of PC attributable to HFPG has shown substantial growth.^[22]^ These divergent trends may be attributed to increasing exposure to environmental carcinogens, changes in dietary and lifestyle behaviors under the influence of globalization, and the accelerating epidemiological transition in developing countries. Furthermore, limited access to effective screening tools, delayed diagnosis, and under-resourced healthcare systems in lower-SDI settings may also contribute to the observed burden.^[23–25]^

Within this broader regional context, China stood out as the country contributing the largest absolute numbers of deaths and DALYs attributable to HFPG. This is largely due to the country’s vast diabetic population, with approximately 140.9 million individuals, the largest worldwide.^[26]^ The prevalence of diabetes in China has steadily increased over the past 30 years,^[27]^ driven by rapid socioeconomic transitions, urbanization, and industrialization. These changes have led to lifestyle shifts – greater intake of high-fat and high-calorie foods, excessive consumption of low-quality carbohydrates, and reduced physical activity – all of which are major risk factors for diabetes and consequently for HFPG-related pancreatic cancer.^[28,29]^ In addition, systemic challenges in diabetes prevention and management exacerbate this burden. Although initiatives such as the National Demonstration Areas have been implemented since 2010, coverage remains limited, reaching <10% of counties.^[30]^ Awareness of diabetes is still below 45%, and glycemic control rates are only around 50%.^[31–33]^ Moreover, rural and underserved areas face shortages of medical resources and screening programs, leading to delayed diagnosis and treatment. These demographics, behavioral, and healthcare factors collectively explain China’s disproportionate share of the national HFPG-related pancreatic cancer burden and underscore the urgent need for more comprehensive and equitable health strategies.

Beyond geographical and socioeconomic disparities, demographic characteristics, particularly sex and age, further shape the heterogeneity of HFPG-related pancreatic cancer burden. This study explored sex-based disparities in the burden of PC attributable to HFPG, revealing a more pronounced increase in both ASMR and ASDR among males compared to females since 1990. Likely due to synergistic behavioral and biological factors: behavioral risks: males showed higher prevalence of PC-promoting behaviors (tobacco use, alcohol intake, and processed meat consumption^[34]^), compounded by smoking-induced glucose dysregulation^[35]^; biological protection in females: estrogen’s dual role in suppressing tumor proliferation (via p53 pathway^[36]^) and stabilizing glucose metabolism may underlie the lower female incidence. This pattern was particularly pronounced in China, where male predominance in diabetes/HFPG^[35]^ aligns with global behavioral trends, suggesting targeted interventions for high-risk males should prioritize lifestyle modification alongside glycemic control. Our analysis also revealed a pronounced age-gradient in PC burden, with peak mortality occurring at 70 to 74 years (250.25 cases/100,000 person-years at FBG ≥ 10.0 mmol/L).^[37]^ The steepest ASMR increase occurred in nonagenarians (EAPC +3.2%), contrasting with minimal change in 25 to 29 year-olds (EAPC +0.8%).This age-disparity reflects progressive molecular dysregulation: chronic HFPG drives AGE-RAGE axis activation, which amplifies oncogenic signaling (PI3K/AKT/mTOR, MAPK) and stromal remodeling (via NF-κB/TGF-β).^[38,39]^ Senescence-associated declines in antioxidant capacity further potentiate this cascade through: ROS-mediated DNA damage, EMT promotion and Immune microenvironment alteration. The resultant pro-tumorigenic milieu suggests elderly diabetics represent a critical prevention target, particularly given their diminished capacity for glycemic stress adaptation.^[39]^

Therefore, based on the results of this study and the successful experience of other countries, we propose the following targeted measures: for patients over 40 years of age with diabetes, it is recommended that PC markers (such as CA19-9) be included in the existing diabetes follow-up management program; Referring to the results of Verna EC et al,^[40]^ imaging screening for PC in high-risk patients is effective and can identify resectable and curable tumors, so we recommend that noninvasive imaging screening such as endoscopic ultrasound be promoted in high-SDI countries. For low and medium-SDI countries, low-cost glucose tests, such as HbA1c fingerstick blood and clinical symptom scores, should be combined, and a pilot project in Kerala, India, increased screening coverage by 40 percent; Priority should be given to pathological diagnostic equipment in high-burden areas such as Eastern Europe and South Asia, such as ultrasound-guided needle biopsy, such as the “Rapid Diagnosis of PC” in Poland, which shortens the time to diagnose PC to 7 days; In areas such as sub-Saharan Africa, where data is lacking, community registration can be carried out through mobile medical vans to improve the data integrity of PC registries, and Rwanda launched a national cancer registry in 2012 that covers 12 referral hospitals, one of the few successful cases in sub-Saharan Africa.

This study has several limitations. First, it does not consider potential interactions between HFPG and other PC risk factors, such as smoking and alcohol consumption, partly due to the aggregated nature of GBD data, which precludes individual-level analyses. Future prospective studies are needed to incorporate such interactions. Second, limited primary data in low-SDI regions may lead to underestimation of the true burden. Although the GBD framework applies robust modeling strategies, including standardized data processing, spatiotemporal Gaussian process regression, and Bayesian meta-regression (DisMod-MR), residual underestimation cannot be excluded, especially in rural and underserved populations (GBD 2021 Risk Factors Collaborators, Lancet 2024; GBD 2021 Diseases and Injuries Collaborators, Lancet 2024; GBD 2019 Risk Factors Collaborators, Lancet 2020).^[2,41,42]^ Third, disparities in healthcare access and diagnostic capabilities may contribute to underreporting or misclassification of HFPG-attributable PC, with residual biases despite statistical adjustments. Future prospective and region-specific studies are warranted to better capture risk interactions, improve data accuracy, and refine burden estimates.

## 5. Conclusion

This study offers a thorough evaluation of the national, regional, and worldwide PC burden caused by HFPG. The results show that during the previous 3 decades, there has been a continuous rise in PC-related fatalities and DALYs linked to HFPG worldwide. Significant regional differences were noted, with high-SDI regions exhibiting the highest ASMR and ASDR. Among all global regions, East Asia – particularly China – accounted for the greatest absolute burden. These results underscore the urgent need for targeted prevention strategies and region-specific public health interventions to address the rising burden of HFPG-attributable PC and mitigate its impact across diverse populations.

## Acknowledgments

Thanks to the Institute for Health Metrics and Evaluation and the Global Burden of Disease study collaborations.

## Author contributions

**Conceptualization:** Jiaqi Han, Qianping Liang, Chan Liao, Shaoqi Yang, Feifei Chu.

**Data curation:** Yaqi Lv, Hongli Chen, Xiaoyu Lei.

## Supplementary Material


