# Supplementary material for: Global, regional, and national analyses of the burden of pancreatic cancer attributable to high fasting plasma glucose from 1990 to 2021: A longitudinal observational study
Source: Medicine (Baltimore). 2026 Apr 17;105(16):e48315. doi: 10.1097/MD.0000000000048315 (PMC13095271; doi:10.1097/MD.0000000000048315)

Supplementary Table 1. Top 10 countries or territories with the highest number of pancreatic cancer Deaths related to high fasting plasma glucose in 2021.

Location	*10^2 No. (95% UI)
China	26256 [2944-50078]
United States of America	20424 [2523-36719]
Japan	11350 [1384-20309]
Germany	6542 [618-1314]
India	5003 [539-9609]
Russian Federation	4505 [476-8922]
Brazil	4147 [473-7859]
Italy	3609 [406-6741]
United Kingdom	3175 [300-6543]
France	2659 [304-5191]

UI: uncertainty interval. The above data has been adjusted by DisMod MR version 2.1.

Supplementary Table 2 Top 10 countries or territories with the highest number of pancreatic cancer DALYs related to high fasting plasma glucose in 2021.

Location	No. (95% UI)
China	6052.86 [690.71-11585.58]
United States of America	4086.21 [511.03-7312.39]
Japan	1803.82 [221.17-3234.72]
India	1189.18 [129.85-2295.36]
Germany	1166.32 [110.55-2367.6]
Russian Federation	993.96 [105.67-1956.07]
Brazil	923.17 [106.57-1735.43]
Italy	630.21 [71.01-1187.88]
United Kingdom	558.95 [53.72-1146.69]
Turkey	503.78 [58.29-965.77]

DALYs: disability-adjusted life-years. UI: uncertainty interval.The above data has been adjusted by DisMod MR version 2.1.

Supplementary Table 3. Top 10 countries or territories with the highest pancreatic cancer ASMR (per 100 000) attributable to high fasting plasma glucose in 2021.

Location	No. (95% UI)
United Arab Emirates	4.27 [0.53-7.6]
Palau	4 [0.54-7.18]
Czechia	3.9 [0.46-7.5]
Greenland	3.46 [0.32-7.77]
Uruguay	3.45 [0.37-6.96]
Montenegro	3.41 [0.42-6.57]
Hungary	3.41 [0.36-6.68]
United States of America	3.33 [0.41-5.98]
Finland	3.27 [0.36-6.39]
Grenada	3.2 [0.34-6.15]

ASMR: age-standardized mortality rate.UI: uncertainty interval.The above data has been adjusted by DisMod MR version 2.1.

Supplementary Table 4. Top 10 countries or territories with the highest pancreatic cancer ASDR (per 100 000) attributable to high fasting plasma glucose in 2021.

Location	No. (95% UI)
United Arab Emirates	83.95 [10.42-148.53]
Czechia	81.24 [9.53-156.96]
Palau	77.23 [10.52-137.98]
Hungary	74.66 [8.19-146.76]
Greenland	73.04 [6.8-160.08]
Uruguay	71.28 [7.85-142.12]
United States of America	69.45 [8.69-123.97]
Montenegro	69.36 [8.7-132.63]
Bulgaria	68.31 [8.54-134.22]
Grenada	67.98 [7.29-132.32]

ASDR: age-standardized DALY rate. UI: uncertainty interval.The above data has been adjusted by DisMod MR version 2.1.

Supplementary Table 5. Top 10 countries or territories with the highest or lowest EAPC in the ASMR (per 100 000) attributable to high fasting plasma glucose，1990-2021.

Location	No. (95% CI)
Turkmenistan	13.46 [11.79 to 15.15]
Cabo Verde	9.05 [7.31 to 10.82]
Mongolia	8.84 [8.12 to 9.57]
Georgia	8.1 [7.47 to 8.73]
Egypt	7.03 [6.5 to 7.55]
Ghana	5.68 [5.35 to 6.01]
Lesotho	5.58 [5.04 to 6.12]
Azerbaijan	4.69 [4.14 to 5.24]
Uzbekistan	4.61 [4.35 to 4.86]
Paraguay	4.48 [4.1 to 4.87]
Mexico	-0.58[-0.68 to -0.48]
Colombia	-0.26[-0.53 to 0.02]
Burundi	-0.24[-0.48 to -0.01]
Bermuda	0.03[-0.14 to 0.2]
Bahrain	0.08[-0.15 to 0.31]
Singapore	0.1 [-0.1 to 0.3]
Saint Lucia	0.13[-0.08 to 0.34]
Ethiopia	0.28[-0.08 to 0.64]
Rwanda	0.3[-0.07 to 0.67]
Cook Islands	0.3[0.15 to 0.46]

ASMR: age-standardized mortality rate. EAPC: estimated annual percentage change.CI: confidence interval.The above data has been adjusted by DisMod MR version 2.1.

Supplementary Table 6. Top 10 countries or territories with the highest or lowest EAPC in the ASDR (per 100 000) attributable to high fasting plasma glucose, 1990–2021.

Location	No. (95% CI)
Turkmenistan	13.53 [11.82 to 15.26]
Cabo Verde	9.02 [7.26 to 10.8]
Mongolia	8.79 [8.06 to 9.52]
Georgia	7.65 [7.05 to 8.26]
Egypt	6.85 [6.37 to 7.34]
Lesotho	5.89 [5.35 to 6.45]
Ghana	5.72 [5.39 to 6.04]
Uzbekistan	4.44 [4.21 to 4.68]
Paraguay	4.43 [4.03 to 4.82
Equatorial Guinea	4.35 [4.09 to 4.6]
Mexico	-0.44 [-0.54 to -0.34]
Burundi	-0.43[-0.69 to -0.16]
Colombia	-0.33[-0.61 to -0.04]
Singapore	-0.22[-0.42 to -0.02]
Bahrain	-0.12[-0.31 to 0.07]
Republic of Korea	-0.12 [-0.21 to -0.04]
Ethiopia	-0.07[-0.45 to 0.31]
Bermuda	0.02 [-0.15 to 0.18]
Maldives	0.04[-0.13 to 0.2]
Rwanda	0.04[-0.37 to 0.45]

ASDR: age-standardized DALY rate. EAPC: estimated annual percentage change. CI: confidence interval.The above data has been adjusted by DisMod MR version 2.1.

Supplementary Figure S1. Clustering of Trends in HFPG-Attributable Pancreatic Cancer ASMR by Country, 1990-2021

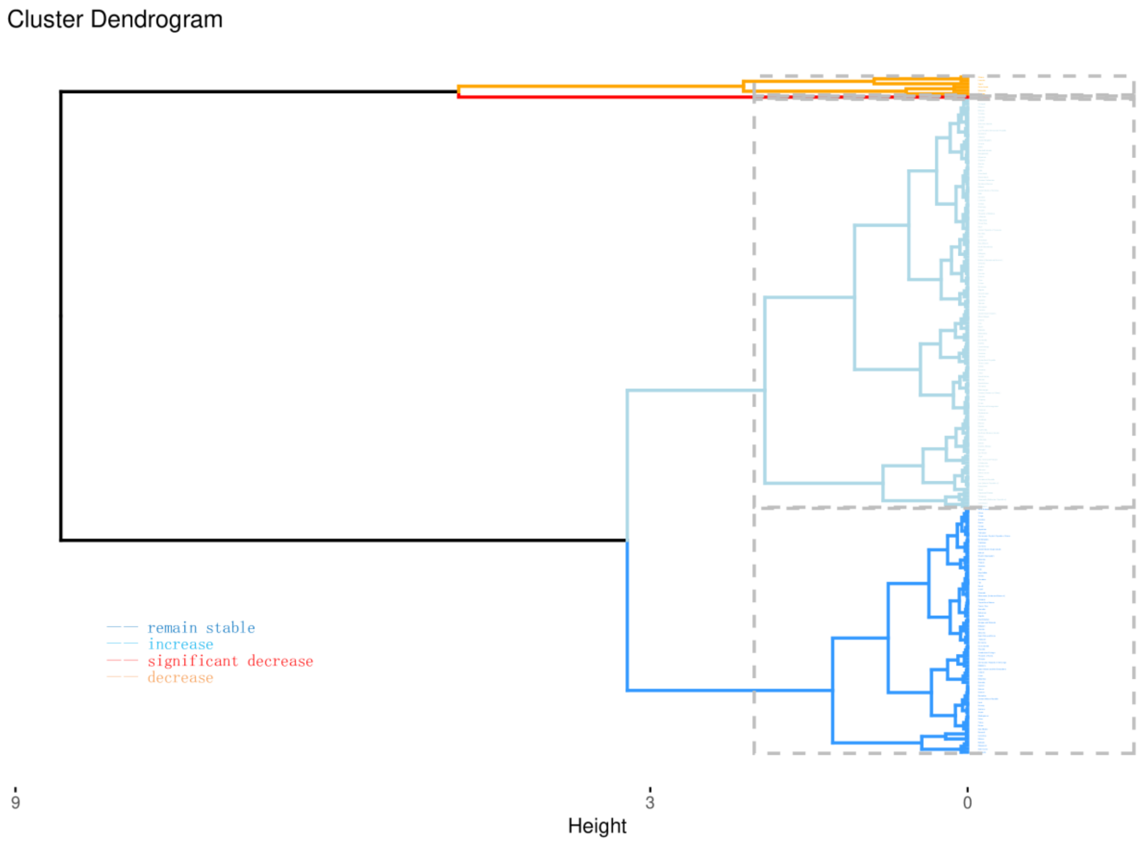

Supplement: Supplementary file 1 [file medi-105-e48315-s001.pdf]
